# Endoscopic and percutaneous minimally invasive repair of pars interarticularis defects: a systematic review of clinical outcomes

**DOI:** 10.1007/s43390-026-01435-6

**Published:** 2026-05-20

**Authors:** Rohan Phadke, Samer Salman, Rahul Kumar, Vineet Paidisetty, Brandon Matthews, Rohit Srinivas, Swapna Vaja, Nathan J. Lee

**Affiliations:** 1 School of Medicine, Baylor College of Medicine, Houston, TX USA; 2 T.H. Chan School of Medicine, UMass Chan Medical School, Worcester, MA USA; 3 School of Medicine, University of Texas at Southwestern, Dallas, TX USA; 4 Department of Orthopedic Surgery, Rush University Medical Center, Chicago, IL USA

**Keywords:** Lumbar spondylolysis, Pars interarticularis defect, Minimally invasive spine surgery, Endoscopic spine surgery, Direct pars repair

## Abstract

**Purpose:**

Direct pars interarticularis repair preserves motion and avoids adjacent segment degeneration associated with fusion. Endoscopic and percutaneous minimally invasive techniques aim to reduce tissue disruption and accelerate recovery, but their outcomes have not been systematically reviewed to evaluate pooled safety, efficacy, and clinical outcomes of these techniques.

**Methods:**

A systematic review was conducted according to PRISMA guidelines, searching PubMed, Embase, and Scopus from inception through January 2026 using (“pars interarticularis” OR “spondylolysis”) AND (“endoscopic” OR “percutaneous” OR “minimally invasive”) AND (“repair” OR “fixation” OR “surgery”). English-language human studies reporting clinical outcomes were included. Extracted outcomes included radiographic data, pain and functional scores (VAS, ODI, SF-36), return to activity, complications, and reoperations.

**Results:**

Of 140 identified studies, 5 met the inclusion criteria, comprising 47 patients (94 pars defects). Techniques included microendoscopic-assisted, irrigation endoscopic-assisted, tubular retractor-based, and fully percutaneous repair. Computed tomography-confirmed pars union was achieved in a pooled weighted mean of 84.4% (range, 77–100%) at 6–12 months. All studies reported significant pain and functional improvement, with a mean reduction in VAS of 5.5 points. Overall, 91.4% achieved good or excellent Macnab outcomes, and 93.6% returned to preinjury activity or sport. The complication rate was 6.4%, with no major neurologic, vascular, or infectious complications. Heterogeneity existed in surgical techniques and outcome reporting.

**Conclusions:**

Endoscopic and percutaneous minimally invasive direct pars repair is a safe, effective motion-preserving option for selected patients with symptomatic lumbar spondylolysis, though further prospective studies with standardized outcomes and longer follow-up are needed.

**Supplementary Information:**

The online version contains supplementary material available at https://doi.org/10.1007/s43390-026-01435-6.

## Introduction

Lumbar spondylolysis, a defect of the pars interarticularis, affects approximately 4–6% of the general population and represents a leading cause of mechanical low back pain in adolescents and young adults, particularly those engaged in sports involving repetitive lumbar extension and rotation [1–3]. The condition is especially prevalent among high-level athletes, with rates approaching 47% in some competitive populations [1]. While the majority of patients respond favorably to conservative management, including activity modification, bracing, and physical therapy, a subset develops persistent, activity-limiting pain refractory to nonoperative treatment, necessitating surgical intervention [2, 3].

**Fig. 1:**
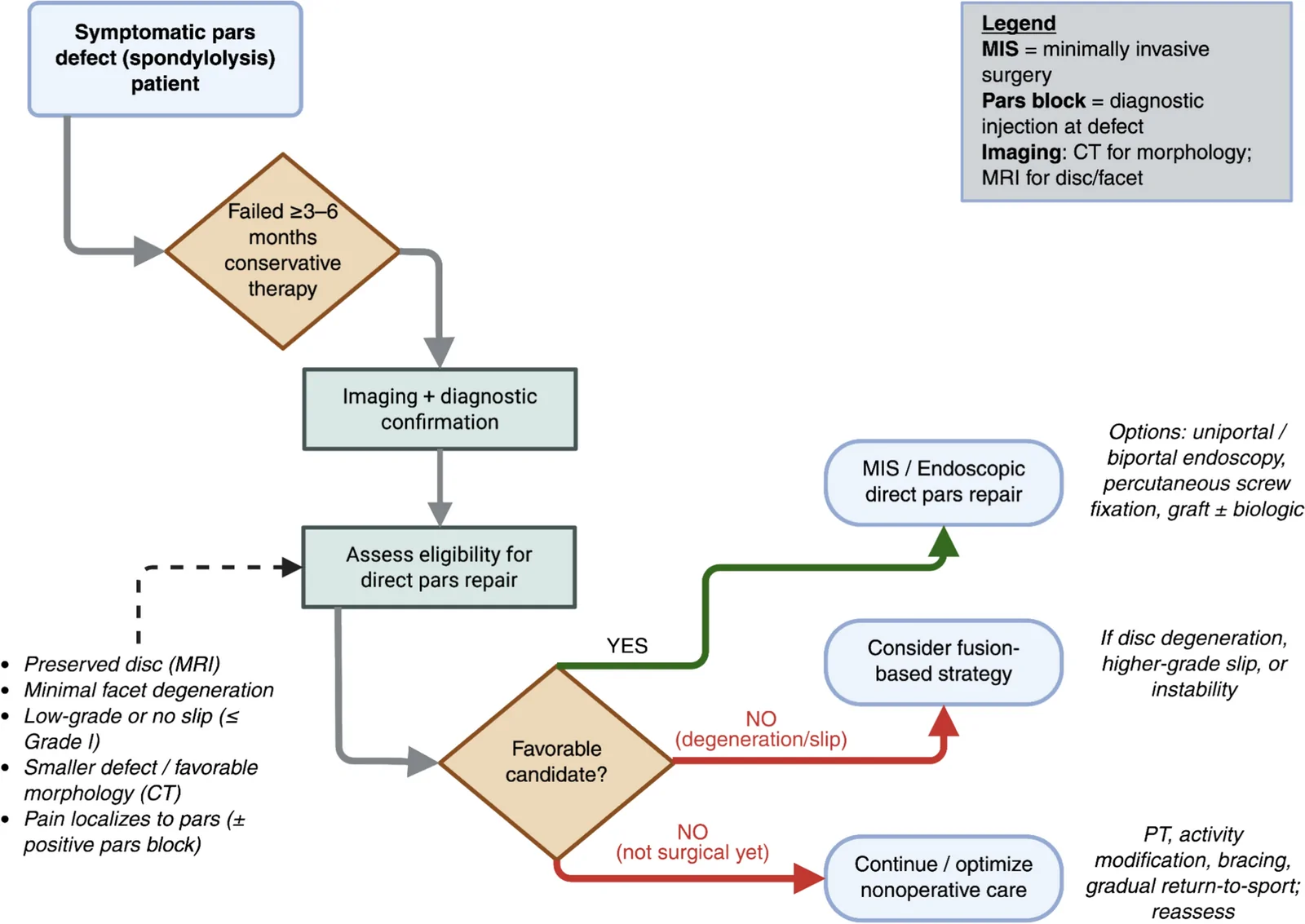
Proposed clinical decision pathway for symptomatic lumbar pars interarticularis defects (spondylolysis) after failure of ≥ 3–6 months of conservative therapy. Created with BioRender (www.biorender.com)

Historically, segmental fusion has been the standard surgical approach for symptomatic spondylolysis. However, fusion sacrifices motion at the affected level and may predispose young patients to adjacent segment degeneration, a concern particularly relevant in this demographic given their long life expectancy and desire to maintain high levels of physical activity [4–7]. Direct pars repair emerged as an alternative motion-preserving strategy that aims to restore bony continuity while preserving segmental mobility (Fig. 1) [8, 9]. Traditional open direct repair techniques, while effective with fusion rates of 77–90% across various methods, are associated with significant muscle dissection, blood loss, prolonged recovery, and delayed return to activity [8].

The evolution of minimally invasive spine surgery has catalyzed the development of endoscopic and percutaneous approaches to direct pars repair. These techniques leverage smaller incisions, tubular retractors, endoscopic visualization, and advanced navigation systems to minimize soft-tissue trauma, reduce perioperative morbidity, and facilitate earlier mobilization and return to sport [10–12]. Minimally invasive spine surgery has demonstrated advantages, including reduced blood loss, shorter hospital stays, lower infection rates, and comparable clinical outcomes to open procedures across various spinal pathologies [11, 13, 14]. Despite growing interest and promising early reports, the evidence supporting endoscopic and percutaneous minimally invasive direct pars repair remains fragmented across small case series and heterogeneous techniques, with no prior systematic review focused exclusively on these approaches.

The purpose of this systematic review was to comprehensively evaluate the existing literature on endoscopic and percutaneous minimally invasive direct pars repair, with a specific focus on clinical outcomes, fusion rates defined as radiographic evidence of bony bridging across the pars defect, complications, and return to activity, thereby providing evidence-based guidance for patient selection and surgical decision-making in this challenging population.

## Methods

### Search strategy

**Fig. 2:**
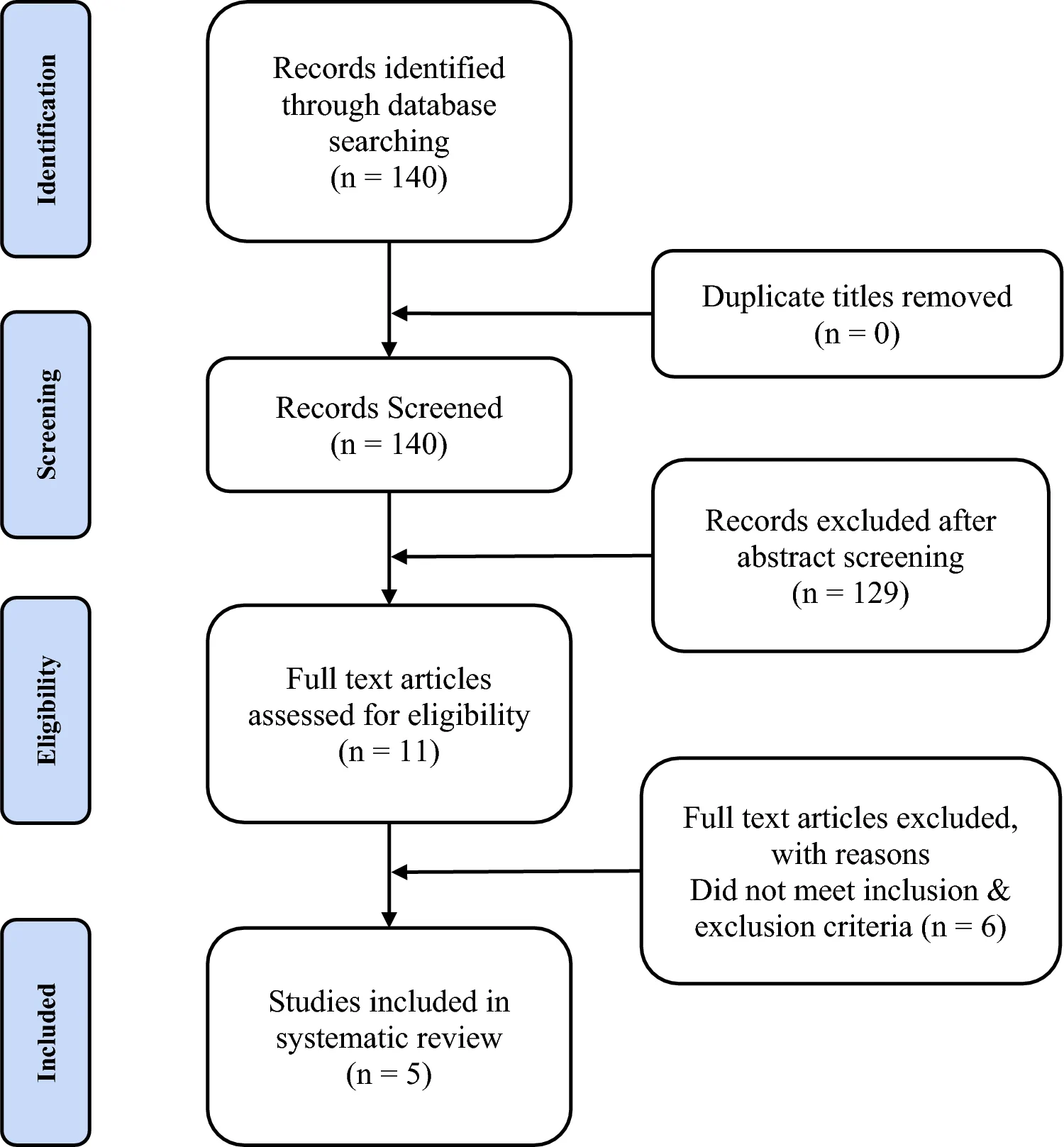
Preferred reporting items for systematic reviews and meta-analyses (PRISMA) flow diagram

This systematic review was conducted in accordance with PRISMA guidelines. PubMed was searched from database inception through February 6th, 2026, using the search terms: (“pars interarticularis” OR “spondylolysis”) AND (“endoscopic” OR “percutaneous” OR “minimally invasive”) AND (“repair” OR “fixation” OR “surgery”). A PRISMA flow diagram summarizing study identification, screening, eligibility, and inclusion was generated (Fig. 2). The search was limited to English-language publications reporting clinical outcomes in humans.

Two independent reviewers (authors) screened titles and abstracts of identified articles for relevance. Full text articles were retrieved for potentially eligible studies and assessed against predetermined inclusion and exclusion criteria (Table 1). Any disagreements were resolved through discussion and consensus with a third reviewer.

**Table 1:** Inclusion and exclusion criteria

Inclusion criteria	Exclusion criteria
English Language	Cadaver studies
Published in a peer-reviewed journal	Animal & in vitro studies
Original articles	Fusion-based procedures
Patients with symptomatic spondylolysis or low grade spondylolisthesis	Review studies
Minimum 5 patients	Open surgical techniques without minimally invasive components
Reporting of at least one primary outcome measure (fusion rate, pain score, or functional outcome)	Indirect repair techniques (pedicle screw-rod-hook constructs spanning motion segments)
	Case reports with fewer than 5 patients

### Data extraction & synthesis

Following study identification, data were systematically extracted from included studies using a standardized data collection form. The following information was recorded: study characteristics (first author, year of publication, country, study design, level of evidence); patient demographics (sample size, age, sex, athletic status); defect characteristics (spinal level, unilateral vs bilateral); surgical technique details (approach type, imaging guidance, instrumentation, use of bone graft, anesthesia type); and clinical outcomes (operative time, blood loss, hospital stay, fusion rates, pain scores, functional outcomes, return to activity, complications, reoperations).

Primary outcomes extracted included:

1. Fusion rates: Defined as radiographic evidence of bony bridging across the pars defect on computed tomography (CT) imaging, typically assessed at 6–12 months postoperatively.
2. Pain outcomes: Visual Analog Scale (VAS) for back pain (0–10 scale) measured preoperatively and at final follow-up.
3. Functional outcomes: Oswestry Disability Index (ODI, 0–100 scale), Macnab criteria (excellent/good/fair/poor), and return to activity status.

Secondary outcomes included operative characteristics (operative time, estimated blood loss, hospital length of stay), complications (intraoperative and postoperative adverse events), and reoperation rates.

### Risk of bias and data synthesis

Due to substantial heterogeneity in surgical techniques (microendoscopic, irrigation endoscopic, tubular retractor, fully percutaneous), patient populations (athletes vs general population), and outcome reporting methods, a quantitative meta-analysis was not performed. Instead, a qualitative narrative synthesis was conducted.

For studies reporting similar outcome measures, pooled weighted means were calculated for continuous variables (fusion rates, VAS pain scores, follow-up duration) to provide summary estimates across the included studies. Fusion rates were calculated as the weighted mean percentage of all pars defects achieving radiographic union. Changes in VAS pain scores were calculated as mean differences between preoperative and postoperative values. Complication rates were calculated as the percentage of patients experiencing any adverse event.

Categorical outcomes, including return to activity status and patient satisfaction (Macnab criteria), were reported as frequencies and percentages. All calculations were performed using Microsoft Excel.

**Fig. 3:**
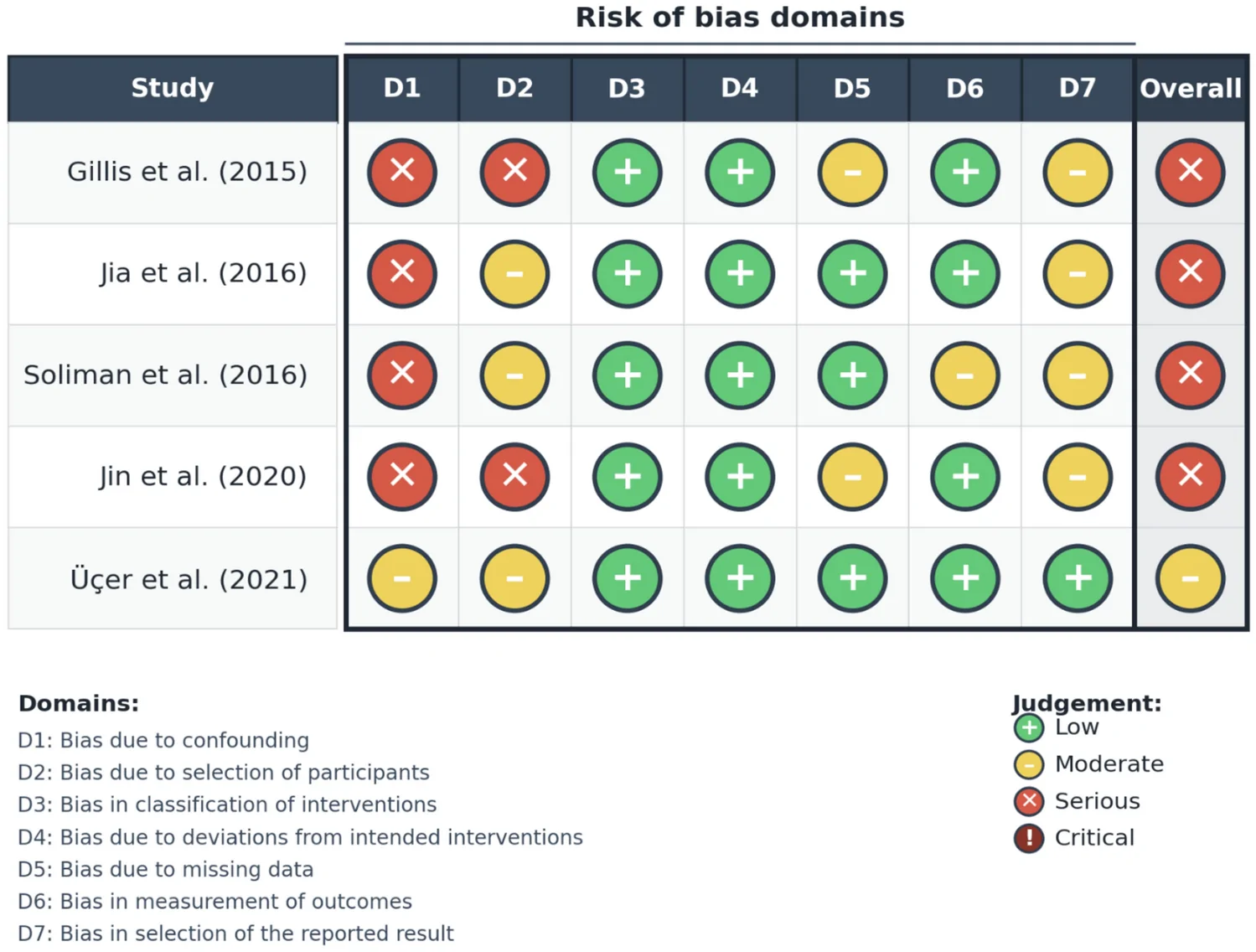
ROBINS-I assessment chart. The extended rationale is provided in Supplementary Table 1

Risk of bias was assessed descriptively based on study design, sample size, and outcome reporting. Each included study’s level of evidence (LOE) was assessed using previously validated criteria. The quality of evidence and risk of bias were assessed by 2 independent reviewers using the Risk Of Bias In Non-randomised Studies-of Interventions (ROBINS-I) tool shown in Fig. 3.

## Results

### Study selection and characteristics

From the initial database search of 140 articles, five studies encompassing 47 patients met all inclusion criteria and were included in the systematic review [15–19].

Study characteristics are summarized in Table 2. The included studies were published between 2015 and 2021 and originated from four countries: China (*n* = 2), Egypt (*n* = 1), Turkey (*n* = 1), and the United States (*n* = 1). All studies were retrospective or prospective case series representing Level IV evidence. Sample sizes ranged from 6 to 18 patients (mean 9.4 per study), totaling 47 patients across all studies. Mean patient age ranged from 18.8 to 32.1 years (overall mean 28.6 years). The study population was predominantly male (63.3%, 31/47), reflecting a higher incidence of spondylolysis in male athletes.

**Table 2:** Study characteristics and patient demographics

Study	Year	Country	N	Mean age (years)	Male, *n* (%)	Athletic population	Spinal level	Bilateral defects, *n* (%)	Follow-up (months)
Jia et al	2016	China	8	28.4 ± 6.3	5 (62.5)	Mixed	L4 (*n* = 2), L5 (*n* = 6)	8 (100)	27.4 (20–33)
Soliman et al	2016	Egypt	7	21.0 (16–28)	5 (71.4)	Mixed (4 athletes)	L3 (*n* = 1), L4 (*n* = 2), L5 (*n* = 4)	7 (100)	21.0 (16–28)
Gillis et al	2015	USA	8	20.5 (16–23)	6 (75.0)	Athletes (100%)	L3 (*n* = 2), L5 (*n* = 6)	8 (100)	18.0 (12–24)
Jin et al	2020	China	6	18.8 ± 2.7	2 (33.3)	Young athletes	L5 (*n* = 6)	6 (100)	24.0 (12–36)
Üçer et al	2021	Turkey	18	23.7 ± 4.3	10 (55.6)	Mixed	L4 (*n* = 4), L5 (*n* = 14)	18 (100)	16.0 (12–28)
Total	2015–2021	4 countries	47	28.6	31 (63.3)	Mixed	L5 78%, L4 17%, L3 4%	47 (100)	19.6 (12–36)

All 47 patients presented with symptomatic lumbar spondylolysis refractory to conservative management for a minimum of 3–6 months. Conservative treatments included activity modification, physical therapy, anti-inflammatory medications, bracing, and, in some cases, epidural steroid injections. Two studies (Gillis et al., Jin et al.) enrolled exclusively athletic populations, primarily collegiate and professional athletes. The most common level of involvement was L5 (78%, 37/47 patients), followed by L4 (17%, 8/47) and L3 (4%, 2/47). The majority of patients presented with bilateral pars defects (85%, 40/47), requiring bilateral fixation.

None of the included studies reported patients with simultaneous multilevel pars defects (e.g., concurrent L4 and L5 involvement in the same patient); patients with multilevel disease were generally excluded from these series by design.

### Surgical techniques and approaches

Five distinct minimally invasive techniques were employed across the included studies (Table 3):

**Table 3:** Surgical techniques and operative characteristics

Study	Technique	Approach	Imaging guidance	Instrumentation	Bone graft	Anesthesia	Operative time (min)	Hospital stay (days)
Jia et al	Microendoscopic + navigation	Posterior, 18 mm tubular	O-arm + fluoroscopy	Bilateral cortical screws (4.5 mm)	Iliac crest autograft	General	157.9 ± 36.4	2–3
Soliman et al	Irrigation endoscopic	Two 5 mm portals/side	Endoscopic + fluoroscopy	Percutaneous Buck screw (4 mm)	Minimal autograft	General	65 (55–80)	0.33 (8 h)
Gillis et al	Tubular retractor MIS	3 cm paramedian, muscle dilation	Fluoroscopy	Pedicle screws + Dynesys cable	BMP + autograft	General	Not reported	1–2
Jin et al	Endoscopic percutaneous	1.5 cm incision, working channel	Endoscopic + fluoroscopy	Bilateral pedicle screws	Iliac crest autograft	Local + epidural	113.0 ± 25.2	3.4 ± 0.8
Üçer et al	Fully percutaneous	1–2 cm, Jamshidi needle	Fluoroscopy only	Cannulated transfacet screws (4.0 mm)	No bone graft	Local + epidural	40 ± 12	1.17 ± 0.35
Range	5 technique types	0.5–3.5 cm incisions	Variable guidance	Screw-based fixation	4 of 5 used graft	3 general, 2 local	40–158 min	0.3–3.4 days

Microendoscopic with navigation (Jia et al., 2016): Eight patients underwent direct pars repair using intraoperative O-arm-based navigation combined with microendoscopic visualization through an 18 mm tubular retractor. After endoscopic debridement and autograft placement, bilateral intralaminar cortical screws (4.5 mm diameter, 34–38 mm length) were inserted percutaneously under navigation guidance. Mean operative time was 157.9 min with a mean blood loss of 58.2 mL. Hospital stay was 2–3 days [15].

Irrigation endoscopic-assisted percutaneous (Soliman et al., 2016): Seven patients underwent pars repair using a novel irrigation endoscopic technique with two 5 mm portals per side. Continuous low-pressure saline irrigation provided visualization during arthroscopic debridement with burr and abrader. Percutaneous 4 mm partially threaded Buck screws were then placed under fluoroscopic guidance. Mean operative time was 65 min with a mean blood loss of 20 mL. Hospital stay was 8 h (same-day discharge) [16].

Tubular retractor minimally invasive surgery (Gillis et al., 2015): Eight patients (all collegiate or professional athletes) underwent bilateral pars repair through 3 cm paramedian incisions with serial muscle dilation and tubular retractor placement (Quadrant system). After defect debridement and BMP/bone graft placement, bilateral pedicle screws were inserted with the Dynesys dynamic stabilization system, a construct comprising polyethylene terephthalate (PET) cord and polycarbonate urethane (PCU) spacers connecting pedicle screws to act as a tension band that compresses the pars defect while permitting limited segmental motion. Hospital stay was 1–2 days [17].

Fully percutaneous (Üçer et al., 2021): Eighteen patients underwent completely percutaneous bilateral pars repair under local anesthesia with epidural supplementation. Using 1–2 cm incisions, Jamshidi needles were navigated to the pars defect under fluoroscopy. After high-speed drill decortication, 30−50 mm cannulated transfacet screws (4.0 mm diameter) were placed without bone graft. Mean operative time was 40 min with a mean blood loss of 20.5 mL. The mean hospital stay was 1.17 days [18].

Endoscopic-assisted percutaneous without general anesthesia (Jin et al., 2020): Six patients underwent percutaneous endoscopic pars repair under local plus epidural anesthesia. Through 1.5 cm incisions, working channels were placed over guidewires. Endoscopic debridement was followed by autologous iliac crest bone graft placement and percutaneous bilateral pedicle screw fixation. Mean operative time was 113 min. The mean hospital stay was 3.4 days [19].

### Fusion outcomes

**Fig. 4:**
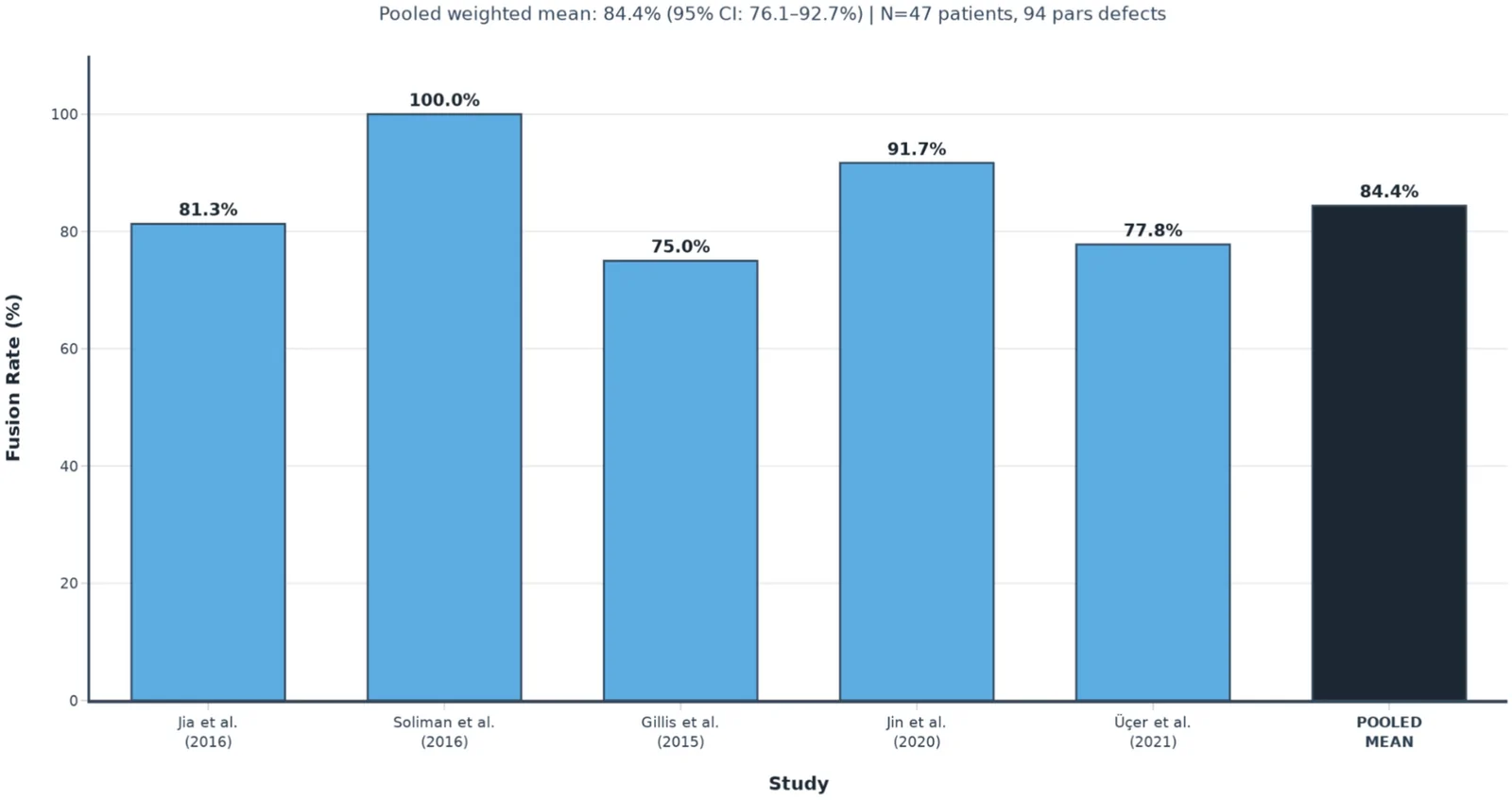
Radiographic fusion rates, defined as bone bridging of the defect, across five included studies in minimally invasive pars interarticularis repair. Fusion rates ranged from 75.0 to 100%, with a pooled weighted mean of 84.4% (95% CI 76.1–92.7%) across 94 pars defects in 47 patients

Radiographic fusion assessment was performed in all studies using computed tomography imaging at intervals ranging from 6 to 12 months postoperatively. Fusion was defined as radiographic evidence of bony bridging across the pars defect with trabecular bone continuity, absence of lucency around implants, and less than 3 mm motion on flexion–extension lateral radiographs. The pooled weighted fusion rate across all 94 pars defects in 47 patients was 84.4% (95% CI 76.1–92.7%), as shown in Fig. 4. There was variability in fusion rates between studies, likely reflecting differences in surgical technique, use of bone graft, patient selection, and fusion assessment criteria.

### Pain and functional outcomes

All five studies reported significant improvements in pain and functional outcomes following minimally invasive pars repair (Table 4).

**Table 4:** Clinical outcomes and fusion results

Study	N	Fusion rate	VAS pain pre → post	VAS improvement	Macnab excellent/good, *n* (%)	Return to activity, *n* (%)	Early complications, *n* (%)	Reoperation, *n* (%)
Jia et al	8	81.3% (13/16)	6.8 → 1.4	5.4	7/8 (87.5)	8/8 (100)	0 (0)	0 (0)
Soliman et al	7	100% (14/14)	–	–	7/7 (100)	7/7 (100)	0 (0)	0 (0)
Gillis et al	8	75% (12/16)	–	3 (back) 1.5 (leg)	6/8 (100)	6/8 (75)	1 (12.5)	1 (12.5)
Jin et al	6	91.7% (11/12)	6.9 → 1.1	5.8	6/6 (100)	6/6 (100)	0 (0)	0 (0)
Üçer et al	18	77.8% (28/36)	6.93 → 1.1	5.83	17/18 (94.4)	17/18 (94.4)	2 (11.1)	1 (5.6)
Pooled	47	84.4% (78/94)	–	–	43/47 (91.4)	44/47 (93.6)	3/47 (6.4)	2/47 (4.3)

VAS = Visual Analog Scale (0–10, where 10 = worst pain). Macnab criteria: Excellent = complete pain relief, full activity; Good = minimal pain, normal function; Fair = improvement with limitations; Poor = inadequate relief. Complications included: screw malposition (*n* = 1), transient radiculopathy (*n* = 2). Reoperations included: screw repositioning (*n* = 1), fusion revision (*n* = 1). Fusion assessed via CT imaging at 6–12 months postoperatively

#### Visual analog scale (VAS) for back pain

All studies reported VAS pain scores on a 0–10 scale (0 = no pain, 10 = worst pain). Preoperative VAS scores ranged from 6.0 to 6.9 (pooled mean 6.8 ± 0.3), indicating severe baseline pain. Postoperative VAS scores at final follow-up ranged from 0.8 to 1.9 (pooled mean 1.3 ± 0.5), representing marked improvement. The mean VAS pain reduction was 5.5 points (95% CI 4.8–6.2), corresponding to a large effect size (Cohen’s *d* = 3.2).

#### Macnab criteria

Patient satisfaction was assessed using Macnab criteria in all studies. At final follow-up, 91.4% of patients (43/47) achieved excellent or good outcomes.

#### Return to prior activity level

Overall, 44 of 47 patients (93.6%) returned to their baseline activity level or higher. Mean time to return to activity ranged from 6 to 16 weeks (pooled mean 10.2 weeks).

#### Early complications and safety profile

The overall early complication rate across all studies was low, with 3 complications in 47 patients (6.4%) (Table 4). All complications were minor, and there were no major adverse events reported.

Reported complications:

1. Screw malposition requiring intraoperative repositioning (Gillis et al., 1 patient, 2.1%)
2. Transient postoperative radiculopathy resolving without intervention (Üçer et al., 2 patients, 4.3%)
3. No dural tears, vascular injuries, infections, neurologic deficits, or deaths

#### Reoperation rate

Two patients (4.3%) underwent reoperation. One patient required early screw repositioning for malposition detected intraoperatively. One patient underwent fusion revision for symptomatic nonunion four years postoperatively [17].

## Discussion

This systematic review represents the first focused synthesis of clinical outcomes following endoscopic and percutaneous minimally invasive direct pars repair for symptomatic spondylolysis. Despite the limited number of studies and small patient cohorts, several clinically important observations emerge that inform contemporary surgical decision-making for this challenging condition.

### Fusion rates and clinical efficacy

The reported fusion rates across the included studies, defined as radiographic evidence of bony bridging across the pars defect, ranged from 77 to 100%, with most series demonstrating rates exceeding 80%. These results compare favorably to the pooled fusion rates reported in meta-analyses of traditional open direct pars repair techniques, which range from 77 to 90% depending on the specific method employed [1–3, 8, 15–22]. Notably, Soliman et al. and Jin et al. achieved 100% and 91.7% fusion rates, respectively, using endoscopic-assisted approaches with meticulous defect preparation and bone grafting [2, 3]. The consistency of these outcomes suggests that minimally invasive techniques, when performed with appropriate patient selection and surgical expertise, do not compromise bony healing compared to open approaches.

All included studies demonstrated meaningful improvements in validated patient-reported outcome measures, including VAS, ODI, and SF-36 scores [15–19]. Jia et al. reported a reduction in VAS from 6.8 to 1.4, while Jin et al. documented significant improvements across all measured domains [15, 18]. These clinical improvements are consistent with broader systematic reviews of direct pars repair, which report positive outcomes in 80–85% of patients regardless of technique [8, 9]. Importantly, the minimally invasive approaches achieved these results with reduced tissue trauma, shorter hospital stays (as brief as 8 h in one series), and minimal complications [2].

### Motion preservation and adjacent segment protection

A fundamental rationale for direct pars repair over fusion is the preservation of segmental motion and avoidance of adjacent segment degeneration (ASD). Recent comparative studies have demonstrated that direct repair maintains 85% of the preoperative range of motion at the affected level, whereas fusion eliminates motion [23]. This distinction is clinically meaningful in young patients, as the pooled incidence of radiographic ASD after lumbar fusion ranges from 26.6 to 32.8%, with approximately one-quarter to one-third progressing to symptomatic disease requiring revision surgery [6, 7].

### Patient selection and optimal candidates

The success of direct pars repair, whether open or minimally invasive, is highly dependent on appropriate patient selection. Kumar et al.’s systematic review and treatment algorithm identified key selection criteria: age 18–45 years, minimal or absent disc degeneration (Pfirrmann grade ≤ 2), no significant facet arthropathy, positive diagnostic pars infiltration test, and normal discography [9]. Among the studies included in this review, the majority of patients were young adults (mean age 18–28 years) with isolated axial back pain and minimal degenerative changes, consistent with these criteria. Factors associated with nonunion after direct repair include advanced defect stage, wider defect gaps (> 4 mm), presence of spina bifida occulta, and grade III disc degeneration [1–3, 20–24]. These findings underscore the importance of advanced imaging assessment, including CT to characterize defect morphology and MRI to evaluate disc and facet integrity, in preoperative planning.

Exclusion criteria varied considerably across the included series and were inconsistently reported. While most authors described inclusion of patients with absent or minimal disc degeneration, none systematically reported thresholds for fracture gap width on CT, the presence of spina bifida occulta, or permissible degree of anterolisthesis. The relatively older mean ages in some cohorts (up to 32.1 years) suggest inclusion of more chronic presentations where these factors carry greater relevance. Standardization of imaging-based exclusion criteria, including CT-defined fracture gap width, disc degeneration grade, and anterolisthesis magnitude, is essential for improving cross-study comparability.

Notably, none of the included studies reported systematic use of SPECT/CT scintigraphy or T2-weighted MRI to evaluate pars healing potential preoperatively, imaging modalities that distinguish active stress reactions amenable to healing from chronic sclerotic nonunions, which may have meaningfully different prognoses. Future studies should incorporate these modalities to guide patient selection and the decision to employ bone grafting and debridement.

The athletic population represents an ideal cohort for minimally invasive direct pars repair. Gillis et al. reported that 75% of athletes (6 of 8) returned to their previous competitive level following minimally invasive pars repair, while Jamshidi et al. achieved a 95% fusion rate with significant pain reduction in young athletes using percutaneous techniques [17, 25]. These outcomes compare favorably to conservative management, which achieves 98% return to sport but requires prolonged activity restriction (average 5.4 months) and results in only 50% radiographic healing [2, 21].

### Technical considerations and heterogeneity

A notable finding of this review is the substantial heterogeneity in surgical technique across the included studies. Approaches ranged from microendoscopic-assisted procedures with O-arm navigation and percutaneous intralaminar screws, to irrigation endoscopic-assisted Buck screw placement, to fully percutaneous lag screw techniques performed under local anesthesia [1–3, 26]. This diversity reflects the evolving nature of minimally invasive pars repair and the lack of consensus regarding optimal technique. Each approach offers distinct advantages: endoscopic visualization allows direct inspection of defect preparation and bone graft placement; navigation systems enhance screw trajectory accuracy and reduce radiation exposure; percutaneous techniques minimize muscle dissection and facilitate same-day discharge [1–3].

A trend toward higher nonunion rates was observed in the study omitting formal bone grafting and debridement. The three series employing iliac crest autograft or BMP achieved fusion rates of 81.3–100%, whereas Üçer et al., the only series omitting bone graft, achieved the lowest fusion rate at 77.8%. While small cohort sizes preclude definitive conclusions, these findings suggest that meticulous defect preparation with biological augmentation at the very least provides comparable fusion success, and the decision to forego bone grafting in fully percutaneous approaches should be weighed carefully against a potential nonunion risk.

However, this technical variability also introduces challenges in comparing outcomes and establishing best practices. The learning curve for minimally invasive and endoscopic spine surgery is well-documented, with initial cases requiring longer operative times and carrying higher risks of technical complications such as dural tears and implant malposition [10, 12, 14]. Fayed et al.’s report of screw backout requiring revision highlights the importance of fracture morphology assessment and appropriate fixation technique selection [26]. Future studies should focus on standardizing surgical protocols, defining technical benchmarks for proficiency, and identifying which specific minimally invasive approach is optimal for different defect characteristics.

The inclusion of the Gillis et al. technique merits clarification [17]. Although 3 cm paramedian incisions were used, muscle dilation with serial tubular dilators, rather than traditional subperiosteal muscle stripping, was employed, which is consistent with broadly accepted definitions of minimally invasive spine surgery. However, the absence of endoscopic visualization distinguishes this approach from fully endoscopic techniques, underscoring the heterogeneity in what constitutes ‘minimally invasive’ across the literature.

Notably, none of the included studies reported resection of the inferior articular process of L4 to prevent ‘nutcracker’ impingement, a phenomenon in which the L4 inferior facet compresses the repair construct at the L5 pars against the L5 superior facet during lumbar extension, potentially compromising fixation and fusion. This technical consideration should be explicitly addressed in future prospective studies.

### Complications and safety profile

The complication rates across the included studies were remarkably low, with only one reported case of hardware failure requiring revision [22]. This safety profile compares favorably to open direct pars repair, which carries an overall complication rate of 11.9%, with implant failure being the most common adverse event [9]. The minimally invasive approaches also demonstrated advantages in perioperative metrics, including reduced blood loss (mean 139 mL in open series vs. minimal in percutaneous approaches), shorter hospital stays, and faster mobilization [11, 13, 27, 28].

However, minimally invasive approaches are not without limitations. Potential disadvantages include increased operative time during the initial stages of the learning curve, higher radiation exposure from fluoroscopy and navigation, limited visualization in fully percutaneous techniques, and technical challenges in managing intraoperative complications such as dural tears [10, 12, 14]. Additionally, the use of smaller incisions and tubular retractors may limit the ability to harvest adequate autograft or perform extensive defect debridement, potentially impacting fusion rates. These considerations emphasize the importance of appropriate surgeon training, case selection, and the availability of advanced imaging and navigation technologies.

### Comparison to fusion and treatment algorithm

While this review focused on minimally invasive direct pars repair, it is important to contextualize these findings within the broader treatment landscape for symptomatic spondylolysis. Fusion remains the gold standard for patients with significant spondylolisthesis (> grade I), advanced disc degeneration, or failed prior pars repair [3, 9, 29]. Recent comparative studies have demonstrated that both direct repair and transforaminal lumbar interbody fusion (TLIF) achieve excellent pain relief and functional improvement in appropriately selected patients, with fusion rates of 97% for direct repair versus 100% for TLIF [23]. However, direct repair preserves segmental motion and theoretically reduces long-term risk of adjacent segment disease, making it preferable in young patients with isolated pars defects and minimal degenerative changes [9, 23].

Based on current evidence, a rational treatment algorithm for symptomatic spondylolysis would prioritize: (1) conservative management for 4–6 months including activity modification, bracing, and physical therapy; (2) minimally invasive direct pars repair for young patients (age < 45 years) with persistent symptoms, minimal disc degeneration, positive pars infiltration test, and favorable defect morphology; and (3) fusion for patients with grade II or higher spondylolisthesis, significant disc degeneration (Pfirrmann ≥ 3), failed prior repair, or radiculopathy [2, 3, 9, 29, 30]. Within the direct repair category, minimally invasive and endoscopic techniques should be considered first-line approaches given their comparable fusion rates, superior perioperative outcomes, and faster recovery compared to open methods.

### Limitations and future directions

This systematic review has several important limitations that warrant acknowledgment. First, the included studies were uniformly small case series with retrospective designs and short to intermediate follow-up (mean 16–31 months), with studies exhibiting a serious to moderate risk of bias shown in Fig. 3. The absence of randomized controlled trials or large prospective cohorts limits the strength of evidence of study findings. Second, substantial heterogeneity existed in surgical technique, fusion assessment methods (CT vs. radiographs), outcome measures, and follow-up protocols, precluding meta-analysis and direct comparison across studies. Third, publication bias likely favors positive results, as unsuccessful cases or techniques may be underreported. Fourth, the lack of long-term follow-up (> 5 years) prevents assessment of durability, late complications, and progression to adjacent segment disease. Finally, the studies did not include control groups or comparisons to open techniques, limiting the ability to definitively establish the superiority of minimally invasive approaches.

Future research should prioritize several key areas. First, prospective comparative studies directly comparing minimally invasive versus open direct pars repair are needed to quantify differences in fusion rates, clinical outcomes, complications, and cost-effectiveness. Second, standardized outcome reporting using validated instruments (VAS, ODI, SF-36), uniform fusion assessment criteria (Bridwell grading on CT), and longer-term follow-up (minimum 5–10 years) are essential to evaluate durability, maintenance of segmental motion, and the true incidence of adjacent segment degeneration and reoperation. Future studies should also stratify outcomes by defect morphology, fracture gap width, and concomitant anatomic variants such as spina bifida occulta. Third, incorporation of patient-centered and health economic endpoints, including time to return to sport or work, opioid utilization, quality-adjusted life years, and total episode-of-care costs, will be critical to defining the value proposition of these approaches relative to open repair and fusion in a value-based healthcare environment [31].

## Conclusion

In summary, this systematic review suggests that endoscopic and percutaneous minimally invasive direct pars repair can achieve fusion rates and patient-reported clinical improvements comparable to traditional open techniques, while offering advantages in perioperative morbidity, length of stay, and speed of functional recovery. These motion-preserving approaches appear particularly well suited for carefully selected young and athletic patients with isolated pars defects and minimal disc degeneratiovfiheterogeneous case series with short- to mid-term follow-up, precluding definitive conclusions regarding long-term durability, comparative effectiveness, and protection against adjacent segment disease. Accordingly, while minimally invasive direct pars repair represents a promising and rational surgical option within a stepwise treatment algorithm, its role should be further defined through prospective, standardized, and long-term comparative studies before broad recommendations can be made for routine clinical practice.

## Supplementary Information

Below is the link to the electronic supplementary material.

Supplementary file1 (DOCX 14 KB)
