# Supplementary material for: Endoscopic and percutaneous minimally invasive repair of pars interarticularis defects: a systematic review of clinical outcomes
Source: Spine Deform. 2026 May 20;14(6):2315–26. doi: 10.1007/s43390-026-01435-6 (PMC13690919; doi:10.1007/s43390-026-01435-6)
Supplement: Supplementary file 1 [file 43390_2026_1435_MOESM1_ESM.docx]

**Supplementals:**

**Supplementary Table 1.** ROBINS-I Assessment Rationale

| **Domain** | **Key Finding** |
| --- | --- |
| D1: Confounding | Serious for 4 studies (no control groups, unable to adjust for age, athletic status, defect characteristics); Moderate for Üçer et al. (largest sample, prospective design) |
| D2: Selection | Serious for Gillis and Jin (exclusively athletes, highly selected); Moderate for remaining studies (single-surgeon series) |
| D3: Classification | Low for all (surgical interventions clearly defined and standardized) |
| D4: Deviations | Low for all (single-surgeon series with consistent technique adherence) |
| D5: Missing data | Moderate for Gillis and Jin (2 patients lost to follow-up); Low for the remaining studies |
| D6: Measurement | Low for 4 studies (validated instruments: VAS, ODI, CT fusion); Moderate for Soliman (radiographic union assessed on X-ray rather than CT) |
| D7: Reported results | Moderate for 4 studies (selective outcome reporting possible); Low for Üçer (prospective, pre-registered outcomes) |
| Overall | Serious for 4 studies; Moderate for Üçer et al. (prospective design, largest cohort, standardized protocol) |
